# Inkjet-Printed Silver Nanowire Ink for Flexible Transparent Conductive Film Applications

**DOI:** 10.3390/nano12050842

**Published:** 2022-03-02

**Authors:** Shuyue Wang, Xiaoli Wu, Jiaxin Lu, Zhengwu Luo, Hui Xie, Xiaobin Zhang, Kaiwen Lin, Yuehui Wang

**Affiliations:** 1 Zhongshan Institute, University of Electronic Science and Technology of China, Zhongshan 528402, China; shuyewang125@163.com (S.W.); 201921030315@std.uestc.edu.cn (X.W.); JIAC13509809967@163.com (J.L.); luozhengwu128@163.com (Z.L.); Xiehuizsedu@126.com (H.X.); zhangxiaobin@redsolar.com.cn (X.Z.); 2 Department of Material and Energy, University of Electronic Science and Technology of China, Chengdu 610054, China

**Keywords:** silver nanowires ink, inkjet printing, flexible conductive film, electrothermal response

## Abstract

The development of flexible transparent conductive electrodes has been considered as a key issue in realizing flexible functional electronics. Inkjet printing provides a new opportunity for the manufacture of FFE due to simple process, cost-effective, environmental friendliness, and digital method to circuit pattern. However, obtaining high concentration of inkjet- printed silver nanowires (AgNWs) conductive ink is a great challenge because the high aspect ratio of AgNWs makes it easy to block the jetting nozzle. This study provides an inkjet printing AgNWs conductive ink with low viscosity and high concentration of AgNWs and good printing applicability, especially without nozzle blockage after printing for more than 4 h. We discussed the effects of the components of the ink on surface tension, viscosity, contact angle as well as droplet spreading behavior. Under the optimized process and formulation of ink, flexible transparent conductive electrode with a sheet resistance of 32 Ω·sq^−1^–291 nm·sq^−1^ and a transmittancy at 550 nm of 72.5–86.3% is achieved. We investigated the relationship between the printing layer and the sheet resistance and the stability of the sheet resistance under a bending test as well as the infrared thermal response of the AgNWs–based flexible transparent conductive electrode. We successfully printed the coupling electrodes and demonstrated the excellent potential of inkjet-printed AgNWs—based flexible transparent conductive electrode for developing flexible functional electronics.

## 1. Introduction

In recent years, printed electronics (PE) have been considered as an alternative to conventional silicon-based technology due to high manufacturing speed, large-area, low cost, environmental friendliness, and particularly applicable to flexible functional devices [1,2,3,4,5,6,7,8,9,10]. PE refers to the electronic product manufacturing technology that functional conductive inks are directly deposited onto substrate to form electronic component or circuit by printed process. Common industrial PE process includes screen printing [6], gravure printing [7], roll to roll printing [8], inkjet printing [10], and so on [11,12,13,14], and have been applied in some fields such as organic electronics [5,6], flexible electronics [2,3,4,11], wearable electronics [12], etc. Among those, inkjet printings particularly attractive in making controllable structure patterned circuits since it offers digital control on the printing process, is easy to integrate into a high throughput, and can be printed for better functional outcome [13,14,15]. In particular, comparing conventional methods, inkjet printing has the advantages of the production of various complex patterns, high material utilization, and low manufacturing cost. Until now, inkjet printing has been applied to some technologically important active components, including organic light-emitting diodes [16], thin-film transistors [17], radio-frequency identification electronic tags [18], etc. 

Generally, inkjet printing process mainly includes the formation and fragmentation of droplet beams, the formation and flight of independent micro-ink droplets, and the deposition and spreading of droplets on the surface of the substrate [19]. Ink droplets deposited on the surface of the substrate in a certain order form a printed pattern. Because the inkjet printing process needs to drop adjustable liquid on the substrate to achieve higher quality and pattern reproducibility, inkjet printing ink is one of the important factors to obtain high-quality patterns, and it is also the key to the popularization and application of ink-jet printing technology [20,21,22,23]. A stable printable conductive ink is usually obtained as conductive nanostructure in a suitable solvent or mixed solvents containing various additives, including dispersant, wetting agent, moisturizer, defoamer, and leveling agent, etc. [24,25]. It should be pointed out that the amount and type of additives in the ink are required to be as small as possible, and they should be removed as much as possible after heat treatment to obtain the required functional properties. Optimization of the ink rheological properties and printing parameters and heat treatment are needed for the realization of printed patterns. Viscosity of ink is an important parameter of all conductive inks, which is closely related to inkjet printing process. Meanwhile, the surface tension, contact angle and pH of conductive ink need to be considered for high-quality printed patterns. In addition, the rheological properties of the inkjet-printed ink are also evaluated by Reynolds number (Reynolds, Re), the Weber number (Weber, We) and Ohnesorge number (Ohnesorge, Oh), which are related to the density, viscosity, and surface tension of the ink [26,27,28].

Until now, researchers have made a lot of efforts in conductive fillers for inkjet printing inks and have achieved fruitful progress [29,30,31,32,33]. Typical conductive fillers mainly include carbon nanotubes, graphene, and metal nanostructures [32,33,34,35]. Among them, silver nanowires (AgNWs) with high electrical and thermal conductivity and flexibility as well as low cost are considered to be the ideal conductive fillers for inkjet printing inks. However, the one-dimensional AgNWs tends to block the nozzles, which is the key problem restricting the application of AgNWs inks. The size of the AgNWs suitable for the inkjet printing should not be long be long, however, AgNWs with high aspect ratio is beneficial to form networks with excellent optoelectronic properties. Therefore, obtaining high-quality inkjet-printed patterns requires a comprehensive consideration of ink formulation, AgNW size, printing parameters, and heat treatment process [35,36,37].

So far, several literatures reported on the inkjet-printed flexible AgNWs conductive films [31,32,33,34]. Lu et al reported that they prepared AgNWs networks as top electrode for semi-transparent organic photovoltaic devices by inkjet printing, but the distribution of AgNWs was not uniform, and agglomerates of AgNWs were observed on the PEDOT:PSS:MoO3 surface [32]. Coleman et al. reported that they fabricated AgNWs pattern on polyethylene terephthalate (PET) surface by inkjet printing, and the conductivity of pattern was 10^5^ S·m^−1^ and the transmittance of ~50% [13]. However, the pattern was opaque and the nozzle blockage occurred during printing. Zhu et al. reported that they fabricated AgNWs patterns on PET substrates by inkjet printer with the nozzle diameter of the printhead of 80 μm and they were still opaque [35]. Maisch et al. reported that they fabricated the AgNWs film (10 μm of AgNW) by inkjet printer with industrial printheads. AgNWs networks displayed a good balance between conductivity and transmittance due to the uniform distribution of AgNWs [36]. In our group, we have formulated a series of AgNWs inks and investigated the effects of the compositions of ink on the viscosity and surface tension of ink and contact angle between the ink droplet and the PET surface without surface treatment and spreading of the ink on the PET substrate after injection [4,10]. Further, we fabricated flexible transparent AgNWs conductive film on PET substrates and investigated the effects of the number printing layer, heat treatment temperature, drop frequency, and number of nozzle on the microstructures and photoelectric properties of AgNWs networks [4,10]. 

In our previous reports, the AgNWs ink was prepared with AgNWs in ethylene glycol and isopropyl alcohol with a volume ratio of 15:10 and the wetting agent. The appropriate AgNWs ink revealed good rheological property and applicability of inkjet printing. However, we found that the nozzles had to be cleaned each four hours to eject smoothly and form the suitable droplets. The main issue is that ethylene glycol is easy to adhere around the nozzle, resulting in the poor ink fluidity and jet behavior of droplets after printing for a long time. In order to address this issue, in this work, we describe the formulation of a stable AgNWs ink based on the low ethylene glycol content. The formulated AgNWs ink has good printing adaptability and print smoothly for at least eight hours. Printed patterns using this ink demonstrate good conductivity and transparency, which can be tuned by varying the printing layer. In order to prove the applicability of the ink, we printed flexible transparent coupling electrodes. The AgNWs ink allows a single-step fabrication of this pattern, without the need for additional etching. 

## 2. Experimental Section

### 2.1. Materials 

Silver nanowires (AgNWs, 10 mg∙mL^−1^) with a diameter of ~20 nm and a length of 2–5 μm dispersed in ethanol was provided by Haitai Naxin Technology (Chengdu) Co., Ltd., Chengdu, China. Ethylene glycol and isopropyl alcohol and absolute ethanol was purchased from Tianjin Yongda Chemical Reagent Co., Ltd., Tianjin, China. Polyethylene terephthalate (PET) films were purchased from Dinglishen New Materials Co., Ltd., Zhongshan, China. Polyether modified polysiloxane (Silcona 137) was purchased from Oncell Co., Ltd., Guangzhou, China. All of the chemicals were used as received. 

### 2.2. Preparation of AgNWs Conductive Ink and Flexible Conductive Films

The AgNWs inks were prepared by taking different amounts of the wetting agent and isopropyl alcohol or absolute ethanol and a fixed amount of AgNWs and ethylene glycol. The designed amount of wetting agent was added into the mixed solution of ethylene glycol and isopropyl alcohol or ethylene glycol and absolute ethanol and magnetically stirred for 5 min. After 15 min of sonication, 1.74 mL of the AgNWs suspension was added into the above solution and magnetically stirred for 5 min. 0.74 mg·mL^−1^ of AgNWs ink was prepared.

**Figure 1:**
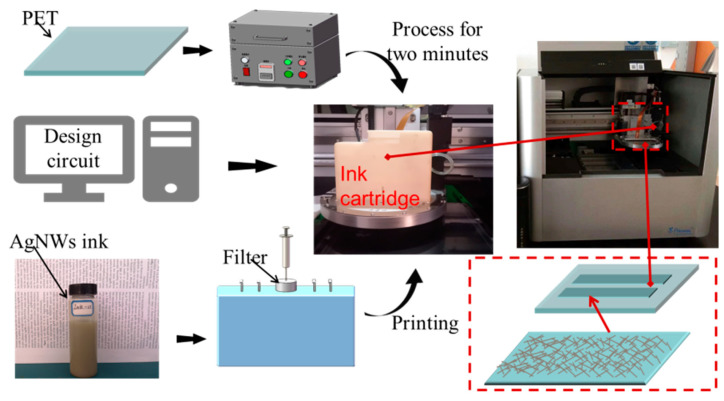
Schematic diagram of the fabrication process of AgNWs-FTCE pattern by inkjet printing.

We designed a square of 1 cm × 1 cm that was arranged in an array of 3 rows and 6 columns by BitsAssembler software of the inkjet printer (Prtronic Scientific 3) that was purchased from Shanghai Mifang Electronic Technology Co., Ltd., Shanghai, China. The AgNWs ink was injected into the cartridge of the inkjet printer after filtered by 0.2 μm polypropylene filter-paper and ink droplets were injected from the nozzle. The inkjet printing parameters for the deposition of the AgNWs network: voltage of 20 V, number nozzles of 8, droplet frequency of 7500 Hz, droplet spacing of 10 μm, the temperatures of the PET substrate and nozzles kept at 40 °C and 35 °C during printing, respectively. After printed one layer, the film was heated at 60 °C for 10 min and then cooled to 40 °C to print the next layer. The AgNWs films with different printed layers were obtained. Figure 1 shows schematic diagram of inkjet printing AgNWs film. The jetting waveform parameters were shown in Figure S1, which was recommended by the equipment manufacturer. In the case of the inkjet printer used in our work, the print waveform parameters chosen depend on the viscosity of the ink. According to the supplier’s suggestion, the ink viscosity should be within the range of 2–10 mPa∙s, and the relevant printing waveform parameters can be selected from the software system of the inkjet printer.

### 2.3. Characterization

The surface structure the printed AgNWs was obtained by using scanning electron microscopy (SEM, Tescan Vega 3, Hitachi Limited, Tokyo, Japan) operated at 10 kV. The viscosity and the surface tension of ink were studied by a digital viscometer (NDJ-1S, Shanghai Qili Scientific Instrument Co., Ltd., Shanghai, China) and an automatic tension meter (JK99C, Shanghai Zhongchen Digital Technology Equipment Co., Ltd., Shanghai, China) respectively. Contact angle (CA) of the ink droplet on the surface of PET was measured by Contact Angle Meter (JC2000C1, Shanghai Zhongchen Digital Technology Equipment Co., Ltd., Shanghai, China). The sheet resistance and transmittance of the film was studied by using a sheet resistance meter (DMR-1C, Nanjing Daming Instruments Co., Ltd., Nanjing, China) and a spectrophotometer (UH415 UV, Beijing Techcomp Scientific Instrument Co., Ltd., Beijing, China). The infrared thermal image of the film was taken by using an infrared thermal imaging camera (UTI160G, range: −20–350 °C, accuracy: ±2 °C, UNI-T China Co., Ltd., Shenzhen, China). A regulated DC power supply (DPS-3010D, Shenzhen Zhaoxin Electronic Equipment Co., Ltd., Shenzhen, China) was used as the driving power supply.

## 3. Results and Discussion

### 3.1. Physical Properties of Sivler Nanowires Ink 

Solvents are the important components of inks and play an important role in the rheological properties and the stability of ink [1]. Ethylene glycol, isopropyl alcohol, absolute ethanol, and water have been reported as suitable solvents for inkjet printing [32,33,34,35,36]. Among them, ethylene glycol is also moisturizer to control the evaporation rate of the solvent and keep the nozzle wet to avoid nozzle clogging due to the high boiling point (197.3 °C) and viscosity (25.66 mPa∙s at 16 °C) of ethylene glycol [4]. In addition, ethylene glycol can also control the rheological properties of inks due to its high viscosity and Newtonian behavior. In our previous work, the formulation of AgNWs ink is followed by taking 15 mL of ethylene glycol, 10 mL of isopropyl alcohol, 10 µL of Silcona 137, and 1 mL of AgNWs suspension, among which ethylene glycol and isopropyl alcohol are cosolvent. To reduce the influence of residual ethylene glycol after heat treatment on the properties of AgNWs film, we optimized the ink formula by decreasing the amount of ethylene glycol and increasing the concentration of AgNWs, that is, the amount of ethylene glycol is 5 mL and the concentration of the AgNWs is 0.74 mg·mL^−1^ (0.38 mg·mL^−1^ in the previous work). in the formulation of ink (0.38 mg·mL^−1^ in the previous work). We investigated the viscosity and surface tension and CA between the droplet and the PET surface and pH value of the inks with the various formulations prepared by adjusting the amount of the isopropyl alcohol or absolute ethanol and the wetting agent, as shown in Table 1.

The photoelectric performance of flexible transparent conductive film depends on the network structure formed by the stacking and overlapping of AgNWs. To avoid nozzle congestion, AgNWs used in the ink must be shortened. In our experiment, AgNW with a diameter of 20 nm and a length of 2–5 µm was used as conductive nano-filler, which are suitable for microelectronic printer inkjet with a nozzle size of 20 µm. In addition, according to the equipment supplier’s recommendation, the viscosity of the ink needs to be controlled in the range of 2–10 mPa∙s to form droplet of suitable shape. Polyether modified polysiloxane (Silcona 137) as the wetting agent and the leveling agent and the defoamer is added into the ink to optimize the rheological properties of the ink.

The viscosity and surface tension of AgNWs ink are two important parameters that affect droplet morphology and size, printability, droplet spreading, and microstructure and performance of the printed film [37,38,39]. Seen from Table 1, the viscosity of ink with the cosolvent of ethylene glycol an isopropyl alcohol is higher than that of the cosolvent of ethylene glycol and absolute ethanol since the viscosity of isopropyl alcohol is higher than that of absolute ethanol. Adding Silcona 137 into the ink with the ethylene glycol and isopropyl alcohol as cosolvent, the viscosity of the ink decreases, while, the viscosity of the ink with the ethylene glycol and absolute ethanol as cosolvent shows little change. A certain amount of the Silcona 137 (5–20 μL) increases the surface tension of the ink with the ethylene glycol and isopropyl alcohol as cosolvent, and the excessive amount of Silcona 137 (25 μL) decreases the surface tension of the ink. However, the surface tension of the ink with the ethylene glycol and absolute ethanol as cosolvent and 5 mL the Silcona 137 increases from 25.19 mN·m^−1^ to 25.40 mN·m^−1^, while, the surface tension of the ink decreases with the increase of the amount of Silcona 137. Overall, Silcona 137 has little effect on the surface tension of both of inks. The as-prepared AgNWs inks in Table 1 were placed in the refrigerator (8 °C) for 40 days and the delamination and deposition of inks were not observed, as shown in Figure S2 of Supplementary Materials. After the inks were dispersed by ultrasound and stirred with a glass rod for 15 min, the inks can still be ejected smoothly, indicating that the inks have good dispersion stability. 

The characteristics of the droplets are usually revealed with Reynolds number (*N_Re_*), Weber number (*N_We_*), and the inverse Ohnesorge number (*N_Re_*), which is designated by Z [28], and they are related to the viscosity and surface tension and density and can be expressed by the following equations:

(1) $$N_{Re}=\frac{v\rho a}{\eta }$$

(2) $$N_{We}=\frac{v^{2}\rho a}{\eta }$$

(3) $$\mathrm{Oh}=\frac{\sqrt{N_{We}}}{N_{Re}}=\frac{\eta }{\sqrt{r\rho a}}$$

wherer *ρ,* υand *η* are the density, dynamic viscosity and surface tension respectively. *r* and a are the velocity of the fluid and characteristic length (refers to the diameter of the nozzle or ink drop), respectively.

Reynolds number and Weber number are not provided because the equipment supplier could not tell us the velocity of the ink. So we simply calculated the inverse Ohnesorge number, namely Z, as shown in Table 1. Reis and Derby reported that stable droplet formation in the process of the ink printing is related to Z values [27,28]. When the Z value is within the limit 1–10, it is conducive to the formation of stable droplets. It can be seen from Table 1 that the Z value of the as-prepared ink is in the range of 5–10, indicating that all of the inks are printable. 

After the ejection of the droplet from the nozzle, the droplet should spread over the substrate surface. The behavior of spreading droplet on a substrate depends on the fluid property, such as surface tension, viscosity, as well as on substrate characteristics [37,38]. The contact angle of the droplet on the surface of substrate is also a very important parameter since it affects the spreading of the droplet on the substrate surface as well as the mutual diffusion between the droplets. Too large a contact angle is not conducive to the mutual *diffusion* and spreading of droplets. Too small of a contact angle easily leads to overflow to affect the accuracy of the printed pattern. The contact angle images of the droplets in Table 1 were shown in Figure S3 of Supplementary Materials. As can be seen from Table 1, as the amount of the Silcona 137 increases, the contact angles of the droplets containing the ethylene glycol-isopropyl alcohol as cosolvent and the ethylene glycol-absolute ethanol as cosolvent on the surface the PET gradually decrease from 29.5° to 17.5° and from 29.0° to 16.5° respectively, indicating the Silican 137 improves the wetting behavior of the droplets on the PET surface. Comparing the parameters of the inks in Table 1, samples of No. 1–6 and No. 11 were used to print on PET substrate.

**Figure 2:**
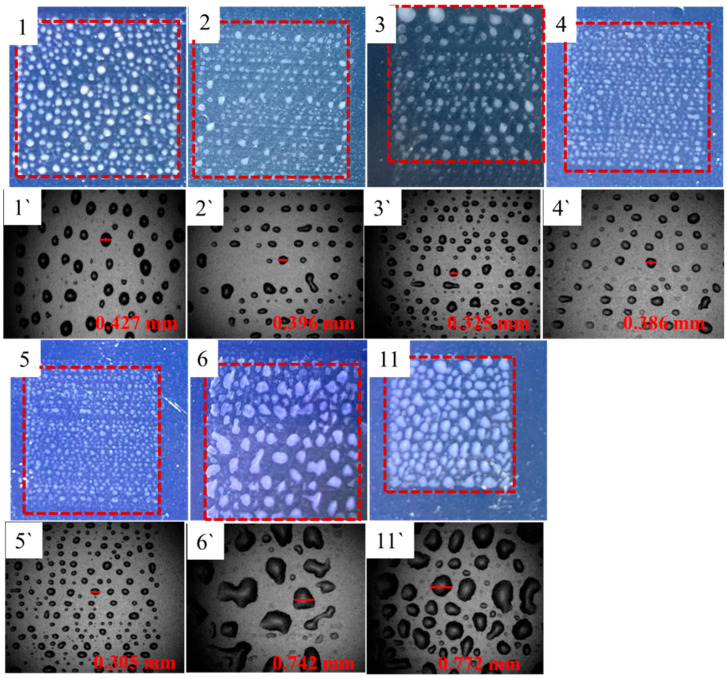
Photographs taken with mobile phone camera (**1**–**6**,**11**) and ink-jet printer’s built-in camera (**1`**–**6`**,**11`**) of the AgNWs inks droplets of No.1 to 6 and 11 in Table 1.

**Figure 3:**
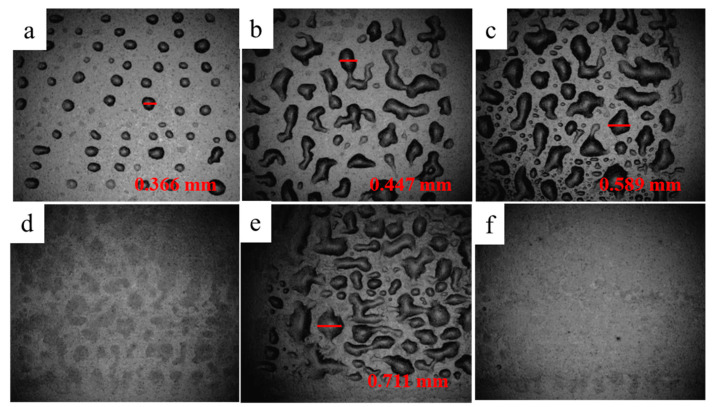
(**a**–**f**) Photographs of 1–6 layers ink droplets printed with the No. 4 ink on the PET substratetaken by the inkjet printer’s built-in camera.

Figure 2 shows photographs of the droplets printed once before heat treatment taken by mobile phone camera (1-6, 11) and ink-jet printer’s built-in camera (1`–6`, 11`), respectively. The size of the brown-red dotted frame in Figure 2 is 1 cm × 1 cm. The values in the photographs taken by the built-in camera are the sizes of the ink droplet in the corresponding photographs. It should be noted that PET substrate was not subjected to any surface treatment. As can be seen from Figure 2, the droplets of No. 1–5 deposited on the PET surface are basically isolated, while the droplets of No. 6 and No. 11 are obvious spreading, diffusion, and some merge into big droplets. The difference of droplet deposition shape maybe related to the impact velocity, viscosity, surface tension, the contact angle, as well as PET surface characteristics. When the droplet is ejected from nozzle to contact PET substrate, it needs to go through the impact, diffusion, recoil, oscillation, until to equilibrium due to the impact of kinetic energy [37,38,39]. The equilibrium shape of the droplet is related to the surface tension. Further, No. 4 ink was used to print 1–6 layers droplets on the PET substrate respectively, as shown in Figure 3. The photographs were taken by the inkjet printer’s built-in camera. After printing twice, it is obvious that the droplets merge and diffuse with each other to form large droplets with different sizes and shapes. As the number of printing layers increases, the number and size of large droplet gradually increase, and small areas of liquid film appear locally. After printing six layers, although there are partial blank areas, the droplets basically converge, diffuse, and spread out to form a liquid film, indicating that the wettability of the droplet on PET surface is not excellent. 

**Figure 4:**
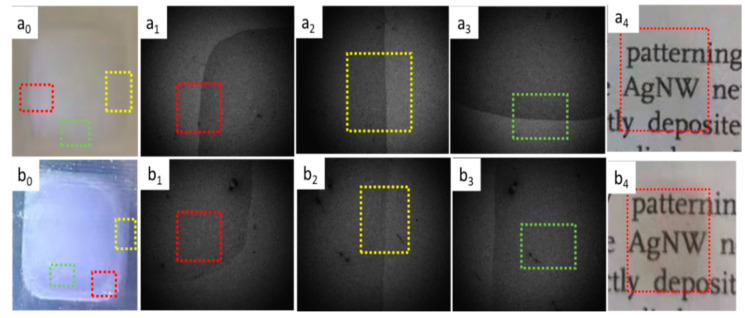
Photopgraphs of one-layer (**a_0_**–**a_4_**) and five-layer (**b_0_**–**b_4_**) ink droplets printed on the PET substrates treated with ultraviolet ozone cleaner for 2 min, respectively, where photographs of the ink droplets before (**a_0_**–**a_3,_b_0_**–**b_3_**) and after (**a_4_**,**b_4_**) heat treatment were taken by mobile phone camera (**a_0_**,**a_4_**,**b_0_**,**b_4_**) and the inkjet printer’s built-in camera (**a_1_**–**a_3_**,**b_1_**–**b_3_**).

The PET substrate surface was treated by ultraviolet ozone cleaner for 2 min and then one-layer (Figure 4a_0_–a_4_) and five-layer (Figure 4b_0_–b_4_) ink droplets were inkjet printed on the above PET substrates with No. 11 ink, respectively. The photographs of the ink droplets before (Figure 4a_0_–b_3_) and after (Figure 4a_4_,b_4_) heat treatment were taken by mobile phone camera and the inkjet printer’s built-in camera. The values in the photographs taken by the built-in camera are the sizes of the ink droplet in the corresponding photograph. The areas of the dotted boxes with different colors in Figure 4a_0_,b_0_ are the location areas of the corresponding color boxes in Figure 4a_1_–a_3_,b_1_–b_3_ respectively. Comparing Figure 3 and Figure 4, the droplets in Figure 4 completely merges with each other and diffuses into a dense liquid film, indicating that the ink droplets have very good wettability on the surface of PET treated by ultraviolet ozone cleaner. It should be pointed out that the liquid film is not a regular square but a rounded square in corners, which may be related to the wettability of ink droplets on the surface of PET. 

### 3.2. Properties of Ink-Jet Printed AgNWs Films

**Figure 5:**
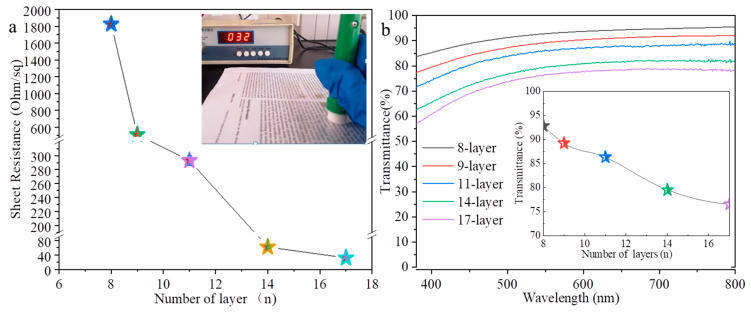
Sheet resistance (**a**) and transmittance (**b**) of the AgNWs films with different layers. The inserts in (**a**,**b**) are the photograph of testing sample and transmittance at 550 nm, respectively.

Figure 5 shows sheet resistances (Figure 5a) and transmission spectra (Figure 5b) of the AgNWs films with different printing layers. The sheet resistances of the 2-layer and 5-layer AgNWs films are not detected, indicating that they are poor conductive. The sheet resistance of the 8-layer film is 1825 Ω∙sq^−1^ and the transparency at 550 nm is 92.8%; the sheet resistance of the 11-layer film decreases dramatically to 291 Ω∙sq^−1^ and the transparency at 550 nm decreases slightly to 86.3%; the sheet resistance of the 11-layer film is 291 Ω∙sq^−1^ and the transmittance at 550 nm is 86.3%. The sheet resistances of films with 14-layer and 17-layer are 62 Ω∙sq^−1^ and 32 Ω∙sq^−1^ and the transparency at 550 nm are 75.5% and 72.5%, respectively. We provided comparison of the sheet resistance and transmittance of inkjet printing AgNWs flexible transparent conductive film in different literatures (as shown in Figure S4 of Supplementary Materials and figure of merit (FOM) of AgNWs films with different printing layers (Figure S5 of Supplementary Materials). FOM of 17-layer of AgNWs film is about 35 and lower than that of previous report, which is related to the small size of AgNWs [13].

**Figure 6:**
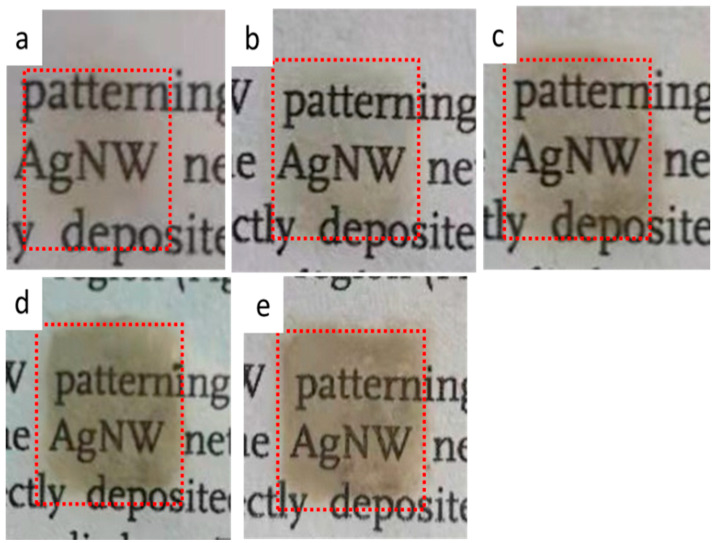
Photographs of AgNWs films with (**a**) 8, (**b**) 9, (**c**) 11, (**d**) 14, (**e**) 17 layers on PET substrates respectively, and the red dotted box is 1 cm × 1 cm in size.

Figure 6 shows the photographs of (Figure 6a) 8, (Figure 6b) 9, (Figure 6c) 11, (Figure 6d) 14 and (Figure 6e) 17 layers on PET substrates respectively, and the red dotted box is 1 cm × 1 cm in size. As the printing layer increase, more of AgNWs are deposited on PET substrate and the film gradually thickens, resulting in a decrease in light transmittance. It is worth pointing out that we did not observe obviously accumulation of AgNWs in 8-, 9- and 11-layer of films, indicating that the AgNWs are relatively uniformly distributed on the PET substrate. Meanwhile, the local accumulation of AgNWs can be observed in 14-layer and 17- layer films, respectively.

**Figure 7:**
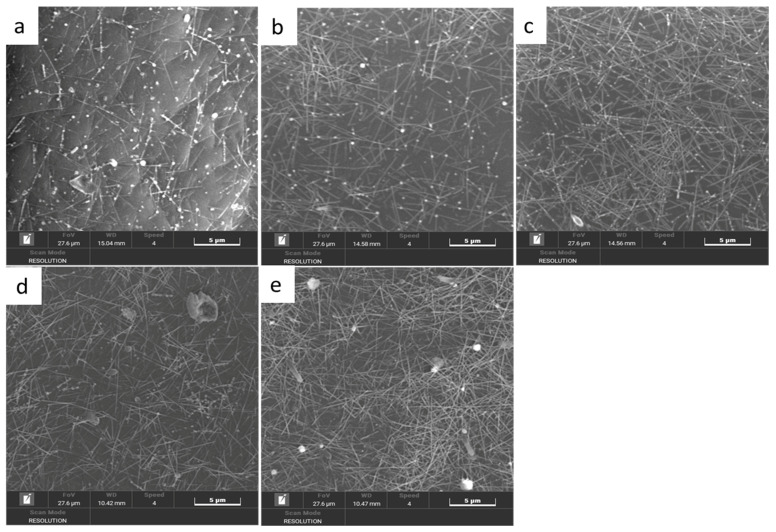
SEM images of of AgNWs films with (**a**) 8, (**b**) 9, (**c**) 11, (**d**) 14, and (**e**) 17 layers of AgNWs film on PET substrate, respectively.

**Figure 8:**
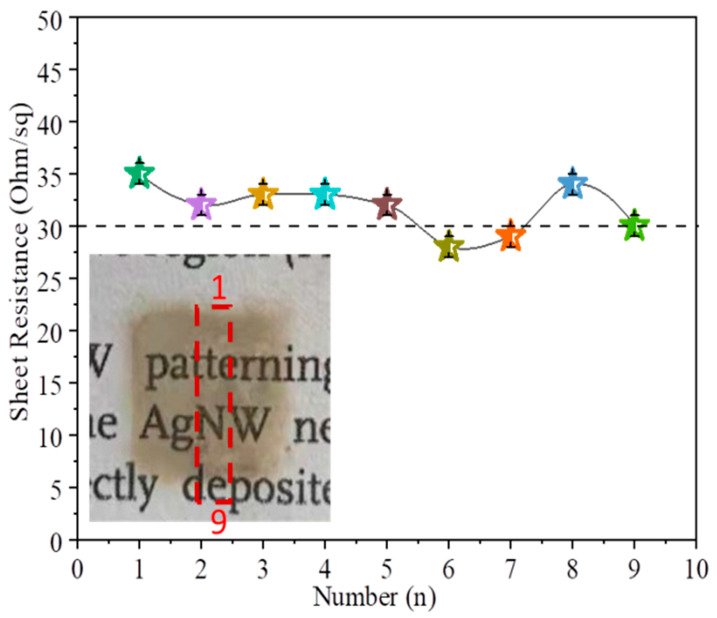
Sheet resistances of nine different points on the 17-layer AgNWs film (in the red dotted box).

Figure 7 shows SEM images of the AgNWs films with (Figure 7a) 8, (Figure 7b) 9, (Figure 7c) 11, (Figure 7d) 14 and (Figure 7e) 17 layers of AgNWs film on PET substrates respectively. It can be seen from Figure 8, the AgNWs are uniformly deposited on the PET substrate as a whole and a small amount of AgNWs overlapped with each other in the 8-layer printing film is observed. The dense networks of the AgNWs overlapped with each other are visible in films with more than 11 layers. 

To further assess the uniformity of the AgNW networks, we selected the middle area of 17-layer AgNWs film (in the red dotted box in Figure 8) and set nine points from top to bottom (the distance between points is the same) to measure the sheet resistance, as shown in Figure 8. Insert is the photograph of sample. The uniformity of the distribution of AgNWs is further evaluated by the change of the sheet resistance. The average sheet resistance of nine points is 31.8 Ω∙sq^−1^, and the maximum difference between the sheet resistances is 11.8%, indicating that the uniformity of the distribution of inkjet-printed AgNWs on the PET substrate is quite excellent. 

**Figure 9:**
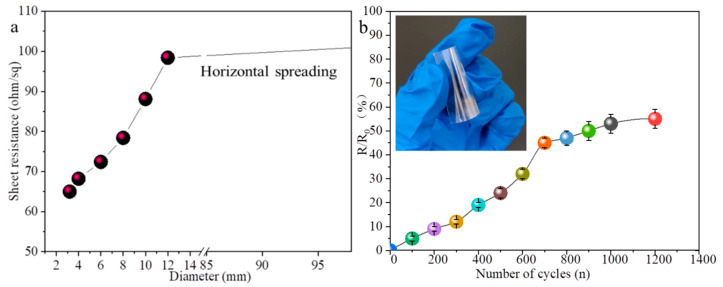
Sheet resistance of the 14-layer AgNWs film on the surface of cylinders of different diameters (**a**) and by different bending cycles of outward bending (**b**). The insert is the testing sample.

The stability of the electrical property of the flexible transparent conductive film is one of the keys to restrict its application. So we studied the stability of the electrical property of the inkjet—printed AgNWs film by coating 14-layer AgNWs film on the surface of cylinders with different diameters (Figure 9a) and repeatedly bending the film outwards by hand (Figure 9b) to test the change of the sheet resistance of film. The insert in Figure 9b is the testing sample. With the the increase of the cylinder diameter, the sheet resistance of the film increases; with the increase of bending cycle in the range of 700 bending cycles, the sheet resistance of film almost increases exponentially, and the relative change in the sheet resistance of the film after 200 bending cycles and 700 bending cycles of outward bending are about 9% and 45% respectively. Increasing the bending cycle from 700 to 1200, the sheet resistance increases gradually, and the relative change in the sheet resistance of the film after 1200 bending cycles is about 55%, indicating that the mechanical stability of the AgNWs film is insufficient. During the bending process, the slippage between the overlapping AgNWs reduces the contact area, so the sheet resistance gradually increases. In addition, the main reason for the obvious increase of the sheet resistance after bending cycle is related to its small size. 

### 3.3. Electrothermal Response of Ink-Jet Printed AgNWs Films

**Figure 10:**
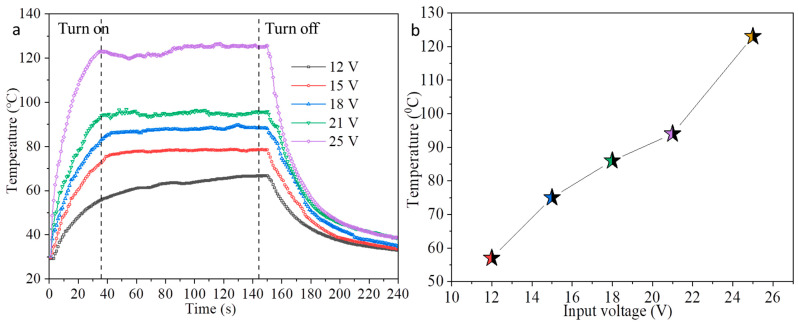
Temperature versus time for 17-layer AgNWs film under input voltage from 12 to 25 V (**a**) and the maximum steady-state temperature of film under different input voltages (**b**).

The electrothermal response performances of the printing film were revealed by applying direct current voltage-stabilized power supply to the film in a laboratory environment. The AgNWs film was made through two clips coated with copper foil that contacted the film edges as electrodes. Figure 10 shows the inkjet- printed 17-layer AgNWs film under the operation for input voltage from 12 to 25 V (Figure 10a) and the maximum steady-state temperature of film at different input voltages (Figure 10b). The input voltage was turned on for 150 s and then turned off. The temperature change of the film was measured by an infrared camera and recorded every second. It can be seen from Figure 10 that the temperature increases almost linearly with time in 20 s, and thereafter increases slowly with time until to reach a steady-state. The steady-state temperature of the film increases as the input voltage increases. When the input voltage increases to 12, 15, 18, 21, and 25 V, the temperatures of the film reach 57 °C, 75 °C, 86 °C, 94 °C, and 123 °C, respectively, indicating that the efficient transduction of electrical energy into Joule heating due to the good conductivity of the printed AgNWs film. After about 40 s, the temperature of the film reaches a stable state under the input voltage from 12 to 25 V, demonstrating fast electrothermal response and suitable for applications in the field of the fast temperature switching with low input voltages. 

### 3.4. Applications of Ink-Jet Printed AgNWs Films

**Figure 11:**
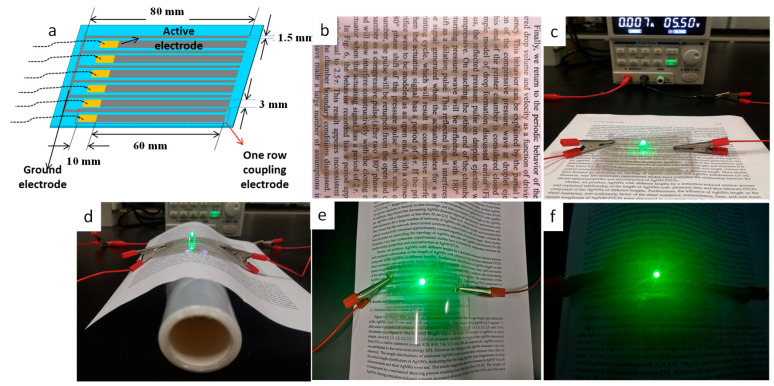
Photographs of designed (**a**) and printed (**b**) coupling electrodes and the coupling electrodes and the LED bead assembled into a circuit in horizontal spreading (**c**) and various bending states (**d**–**f**) after applied direct current (DC) voltage of 5.5 V.

In order to evaluate the practicability of inkjet-printed AgNWs film, we designed (Figure 11a) and printed (Figure 11b) coupling electrodes with 14-layer AgNWs film. The coupling electrodes and the light emitted diode (LED) bead (0.2 W per) were assembled into a circuit (Figure 11c). Seen from Figure 11b–f, the coupling electrodes show a good shape and light transmittance and the LEDs bead can work well in horizontal spreading and various bending states after applied direct current (DC) voltage of 5.5 V, indicating that the well-defined flexible transparent AgNWs conductive pattern can be obtained by inkjet printing. Of course, further improvements, including improving accuracy of line and photoelectric properties, are still necessary. 

## 4. Conclusions

In summary, a low viscosity and high concentration of inkjet-printed AgNWs ink was prepared. The surface tension, viscosity, contact angle, as well as droplet spreading behavior of the inks with different components were discussed. The experimental results demonstrated that the viscosity of the ink is mainly solvent-related, and the spreading behavior of the droplets is mainly related to the wetting agent and the PET substrate surface characteristics. The formulated AgNWs ink has good printing adaptability and print smoothly for at least eight hours. The sheet resistance of the 8-layer of AgNWs film is 1825 Ω∙sq^−1^ and the transparency at 550 nm is 92.8%; the sheet resistance and the transparency at 550 of the printed 14-layer and 17-layer of AgNWs films are 62 Ω∙sq^−1^ and 32 Ω∙sq^−1^ and 79.5% and 76.5%, respectively. The bending test reveals that the relative change in the sheet resistance of the film after 200 and 1200 bending cycles are about 9% and 55% respectively due to the short size of the AgNWs. However, the film shows rapid electrothermal response performance under the input voltages from 12 to 25 V. The maximum steady-state temperature of the film at 12 and 25 V reach 57 °C and 123 °C respectively after 40 s or so. In order to prove the applicability of the ink, we printed flexible transparent coupling electrodes. The AgNWs ink allows a single-step fabrication of coupling electrodes, without the need for additional etching. It promotes inkjet-printed AgNWs-based flexible transparent conductive film becoming an important addition to the current technology in the printing electronics industry.

## Figures and Tables

**Table 1:** Properties of the AgNWs inks with different formulations (at 28 °C).

No.	Isopropyl Alcohol (mL)	Absolute Ethanol (mL)	Silcona 137 (μL)	Viscosity (mPa·s)	Surface Tension (mN·m^−1^)	Contact Angle (°)	pH	Z
1	15	0	0	4.1	23.40	29.5	7.84	5.28
2	15	0	5	3.6	24.53	23.5	7.56	6.15
3	15	0	10	3.4	24.65	21.5	7.47	6.53
4	15	0	15	3.5	24.69	20.5	7.84	6.35
5	15	0	20	3.5	24.19	19.5	8.14	6.28
6	15	0	25	3.6	21.20	17.5	7.93	5.72
7	0	15	0	2.3	25.19	29.0	8.08	9.76
8	0	15	5	2.3	25.40	26.5	8.07	9.80
9	0	15	10	2.4	24.45	23.0	8.13	9.21
10	0	15	15	2.3	24.19	21.5	8.12	9.56
11	0	15	20	2.3	24.65	18.5	7.94	9.65
12	0	15	25	2.2	21.03	16.5	7.22	9.32

## Data Availability

Not applicable.

## Supplementary Materials

The following are available online at: https://www.mdpi.com/article/10.3390/nano12050842/s1, Figure S1: Jetting waveform parameters and voltage; Figure S2: Photographs of the as-prepared AgNWs inks in Table 1 placed in the refrigerator (8 °C) for 0, 3, 15 and 40 days, respectively; Figure S3: Photographs of Contact Angle of AgNWs inks shown in Table 1; Figure S4: Relationship between sheet resistance and transmittance of inkjet printed silver nanowires flexible transparent conductive film in different literatures; Figure S5: FOM of AgNWs films with different printing layers.
