# Supplementary material for: Inkjet-Printed Silver Nanowire Ink for Flexible Transparent Conductive Film Applications
Source: Nanomaterials (Basel). 2022 Mar 2;12(5):842. doi: 10.3390/nano12050842 (PMC8912571; doi:10.3390/nano12050842)
Supplement: Supplementary file 1 [file nanomaterials-12-00842-s001.zip › nanomaterials-1529982-supplementary.pdf]

*nanomaterials*

*Supplementary Materials*

# Inkjet-Printed Silver Nanowire Ink for Flexible Transparent Conductive Film Applications

**Shuyue Wang [1,†], Xiaoli Wu [1,2,†], Jiaxin Lu [1], Zhengwu Luo [1], Hui Xie [1], Xiaobin Zhang [1], Kaiwen Lin, [1,*] and Yuehui Wang [1,*]**

[1] Zhongshan Institute, University of Electronic Science and Technology of China, Guangdong, Zhongshan 528402, China; shuyewang125@163.com (S.W.); 201921030315@std.uestc.edu.cn (X.W.); JIAC13509809967@163.com (J.L.); luozhengwu128@163.com (Z.L.); Xiehuizsedu@126.com (H.X.); zhangxiaobin@redsolar.com.cn (X.Z.)

[2] Department of Material and Energy, University of Electronic Science and Technology of China, Chengdu 610054, China

* Correspondence: wyh@zsc.edu.cn (Y.W.); 201610102331@mail.scut.edu.cn (K.L.); Tel.: +86-760-8832-5402

† These authors contributed equally to this work.

**Citation:** Wang, S.; Wu, X.; Lu, J.; Luo, Z.; Xie, H.; Zhang, X.; Lin, K.; Wang, Y. Fabrication of Inkjet Printability Silver Nanowires Ink with High Concentration and Its Application in Flexible Transparent Pattern Circuit. *Nanomaterials* **2022**, *12*, 842. https://doi.org/10.3390/nano12050842

Academic Editor(s): Christian Falconi and Andrew Pike

Received: 13 December 2021
Accepted: 23 February 2022
Published: 2 March 2022

**Publisher's Note:** MDPI stays neutral with regard to jurisdictional claims in published maps and institutional affiliations.

**Copyright:** © 2022 by the authors. Submitted for possible open access publication under the terms and conditions of the Creative Commons Attribution (CC BY) license (https://creativecommons.org/licenses/by/4.0/).

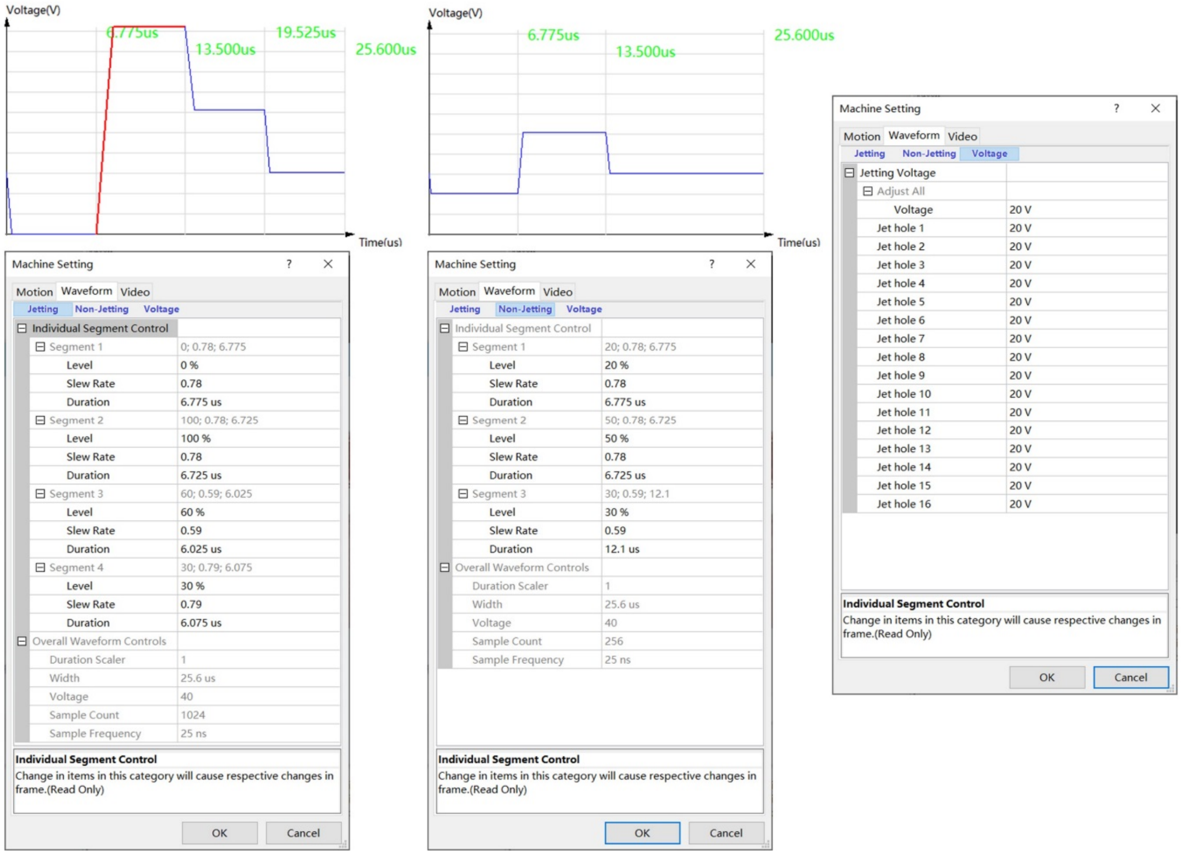


**Figure S1.** Jetting waveform parameters and voltage.

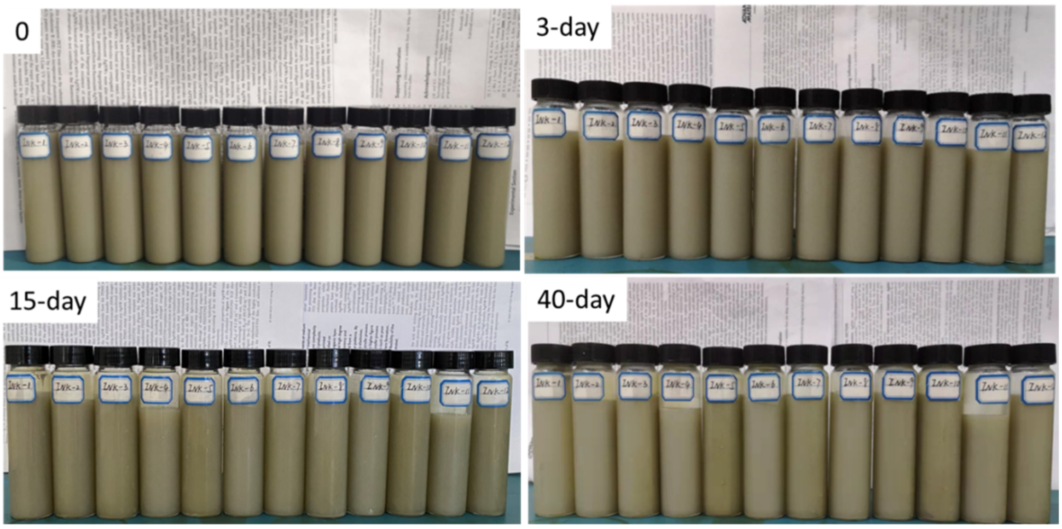


**Figure S2.** Photographs of the as-prepared AgNWs inks in Table 1placed in the refrigerator (8℃) for 0, 3, 15 and 40 days, respectively.

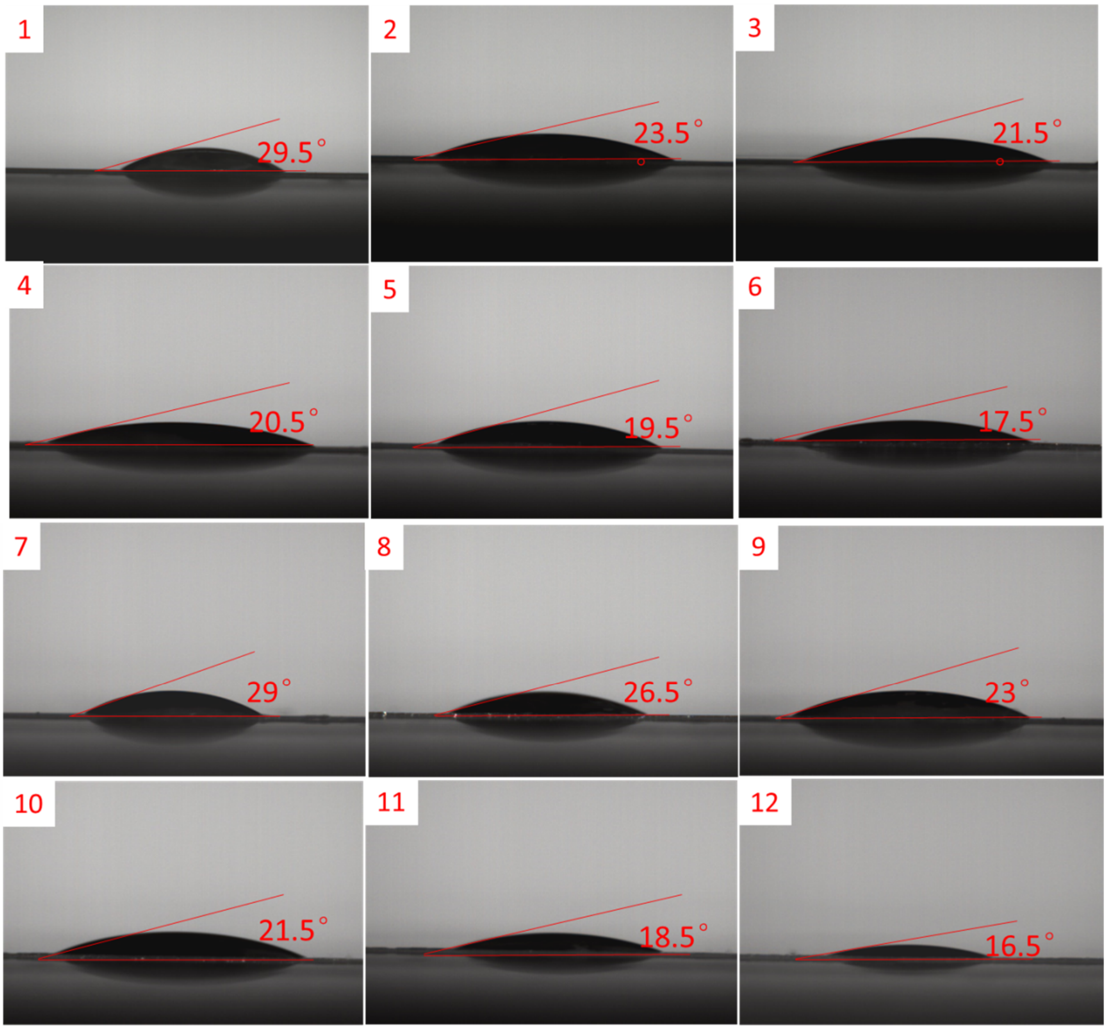


**Figure S3.** Photographs of Contact Angle of AgNWs inks shown in Table 1.

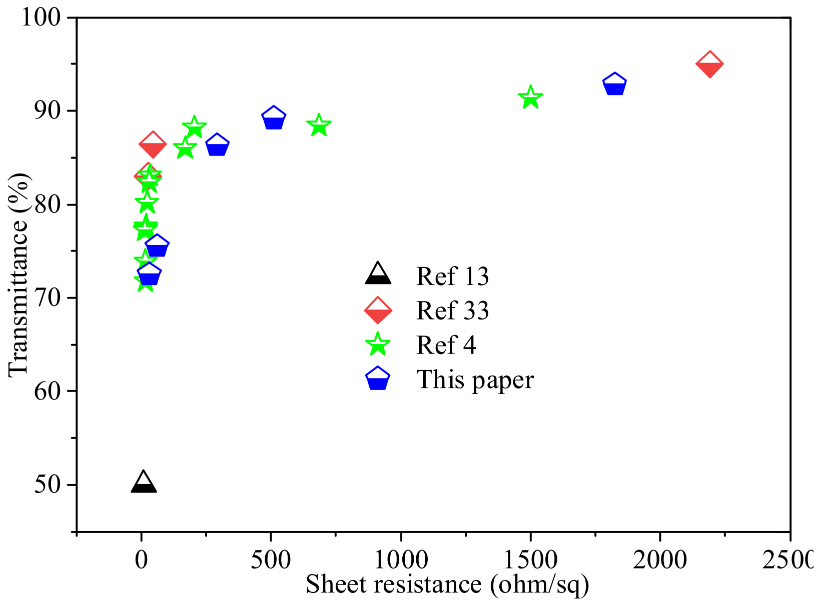


**Figure S4.** Relationship between sheet resistance and transmittance of inkjet printed silver nanowires flexible transparent conductive film in different literatures.

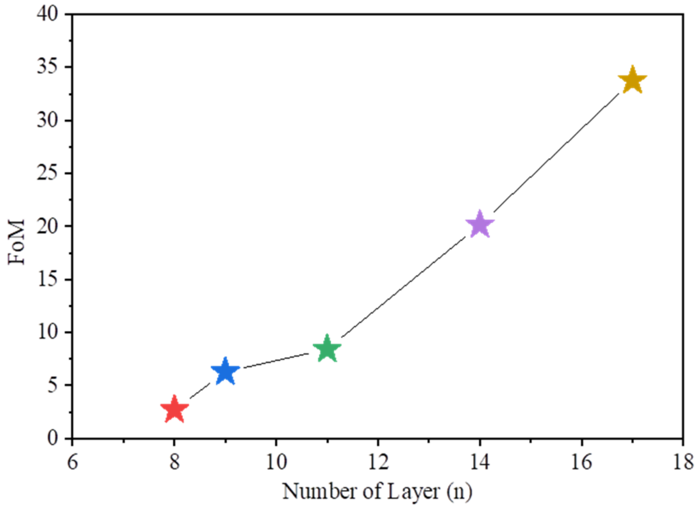


**Figure S5.** FOM of AgNWs films with different printing layers.
